# Lecture on Aconite

**Published:** 1885-02

**Authors:** J. T. Kent

**Affiliations:** St. Louis


### LECTURE ON ACONITE.

PROFESSOR J. T. KENT, M. D., ST. LOUIS.

Aconite is markedly in contrast with Acetic acid. The Aconite condition comes on *suddenly* and *violently*. This is grandly in contrast with Acetic acid. The action of Aconite is first on the brain and spinal cord, and affects most violently the heart and circulatory system. The "red string" that runs through Aconite is its *mental* and *nervous state*. You will hardly cure a case with Aconite unless there are present, viz.: *anxiety* and *fear* of *death;* patient even predicts the *time* he will *die*. Aconite does not correspond to typhoid or puerperal fevers. In typhoid fever the patient has been feeling badly from *four to six weeks*, while the fever of Aconite comes on *suddenly*, with great *thirst* and *anxiety*. Aconite is never indicated in typhoid fever because it cannot produce changes in the blood; it can only produce marked nervous changes. In remittent fever, that comes on *suddenly* in the fore part of the night, from taking cold in cold weather, with *anguish* and *great fear*. In plethoric individuals this condition often occurs during pregnancy. (The weakly, careworn individual is *never* taken *suddenly*.) Rhus is easily distinguished from Aconite. The Rhus patient will tell you it is no use to prescribe for him—he is going to die any way; while the Aconite patient is distressed, and predicts the day or hour.

It is a very bad practice to give Aconite in *every* case of fever. It is not useful in *every* case of inflammation or congestion of the lungs. It is characteristic of Aconite to congest the upper half of the left lung. Because Aconite causes *a* fever, it does not follow that it will cure *every case* of *fever*. The landmarks must be present. Symptoms and keynotes may lead you to find a remedy, but if you prescribe on keynotes alone, you will often fail in practice. You may have a quick, throbbing pulse, thirst, and a septacæmic condition of the blood, which cannot be produced by Aconite. You may treat milk-fever sometimes with Aconite, because you may have in such cases a dry, hot skin, backache, restless anxiety, and anguish. If you have the *mental* state of Aconite *without inflammation*, it is indicated. There is no remedy in the materia medica that has inflammation of the trachea more prominently if you have the mental symptoms, throbbing of the pulse, heat, *fear*, and *anguish* (or no matter where the inflammation is, in trachea, lung, liver, or bladder), than this remedy. If there is inflammation of the neck of the bladder, attended with *great fear*, trembling, anxiety, extreme restlessness, and soreness (Ars. and Rhus have these also), and the patient *predicts* the *day* of *death*, it is Aconite. The delirium of Aconite is rarely attended with unconsciousness. Aconite is a child's remedy; is useful in brain affections of children with violent pain in the head and over-sensitiveness of the eyes; it lies stupid, with vomiting and constipation. You can see the *fear* depicted in the child's countenance, and it has hot skin. Aconite does not affect the parenchyma of the lungs. The pneumonia of children is usually capillary. You need not make physical examination. The objective appearances would lead you to select Aconite—*i. e.*, hot skin, throbbing pulse, *fear* and *anxiety*, thirst. There is no fullness and heaviness in the forehead, as if the brain would start out of the eyes. (Bell. and Bry.; Merc. has it in the winter right in the centre of the forehead; great pressure, as if the brain would be forced out. Throbbing would make you think of Bell. Bry. has a purple face. Merc. patient thinks the pain is in the bone; there is soreness in the bone. The *more sweat* the *more pain*. Merc. is aggravated by *sweating*.) Think of Aconite in all rapid congestions of the brain and other parts of the body, especially where there is anguish and fear. A farmer, lying asleep in the sun, wakes up and has a hot face and *fear depicted* in *his countenance*. (Bell., Cact.-gr., and Glonoine are more commonly used remedies for sunstroke. The patient is delirious and has *no perspiration* —Bell. or Glon. If *head* is *intensely hot* and the *body cold* it calls for Glon. *Glon.* and *Bell.* have an unconsciousness that is not found in Aconite.)

The child takes cold, and convulsions follow as a result. Remember that the onset is *sudden* and with *great violence*. A teething child has convulsions brought on by taking cold. In spasms you would not think of Aconite. You must select a deeper-acting remedy (Ars., Lach., or Sulph., etc.). It would be pardonable to give Aconite if you have its *mental state*. I never found that state of mind present in spasms, however. This anguish comes on *suddenly*. Where there is inflammation of the left lung, the heart is likely to be affected, and it sympathizes with the brain; hence the *mental state*. *Fear* only attends that which is closely connected with the heart.

Aconite produces no formation of pus; this is a negative feature. You may give Aconite where is redness of the mucous membrane, but when pus forms it is not indicated. Aconite produces no gonorrhœa, but some of our wiseacres give it. A

prominent feature of Aconite is *cold sweat* on the *forehead.* Ophthalmia (*without pus*), scalding tears, and general irritability; a flickering before the eyes; pupils contracted and then dilated; eyes red, inflamed, and *burning;* pressing, shooting pains, especially on moving the ball; *no discharge*—if these conditions are brought on by sudden change in the weather, or by *dry, cold winds,* lids are red and swollen; no *purulent formation* nor *exudation.* (*Bry., Sulph.*, and the Kali salts produce exudation.) *Cold water* relieves the *dry heat.* (*Merc.* is a great remedy for this condition of the eye, but it has a purulent exudation and *dry, scaly* appearance around the eyelashes.) The ear symptoms of Aconite belong to brain disturbances; there is aching, tearing, roaring in the ears. You would never think of Aconite for *local* affections of the ear. Nosebleed from fright. (The *fear* of the fright remains—Opium.) Dry or fluent coryza, with violent sneezing; fever, thirst, and restlessness. Coryza with headache. Teeth sensitive to *cold air* in toothache; toothache in sound teeth. The child grinds its teeth during sleep. (*Cina* and *Ars.* Cina is indicated in children affected with worms. In brain disturbances it would be Aconite; in stomach disturbances, *Cina,* and in low remittant fevers, *Ars.*)

Everything tastes *bitter, except water.* The thirst in Aconite is burning and as prominent as in any other remedy, such as *Phos., Acetic acid,* and *Ars.* The taste is bitter and sweetish, or like rotten eggs, flat and nauseous; compels hawking of tough mucus. Dryness of mouth, tongue, and lips. Child while teething will hold cold glass to the gums. (When a toothache is made better by cold think of Ferrum, Phos., Bry., and Magnesia phos.) *Numbness* of *mouth* and *tongue.* Aconite has also this "red string" running through it—*tingling* of *face, fauces,* and *fingers; numbness* of *face, fauces,* and *tongue.* *Aconite Ferox* has a greater numbness in fingers than any other remedy; patient feels as if *he had on gloves.* (*Phos.* also has a *numb* feeling.) *Burning, tingling* sensation along the spine.

STOOL very prominent; watery, black, green, like chopped spinach; corrosive and slimy; small, frequent, and involuntary; stool when passing flatus; before stool *cutting pains, anguish,* and *sweat;* during stool cutting pains, trembling, tenesmus, and sweat; *after stool* relief, except from *anguish.* (*Nux vom.* is the best remedy where there is *relief from stool.* The *Merc.* patient has a "never-get-through" feeling, with aching tenesmus and tormina.) If you have in a child a quick, sharp pulse, dry skin, and bloody mucous discharges, perhaps green with *fear,* it is Aconite. Urging, slimy stool nightly. Bleeding piles—

pressing and stinging in the anus. (Dr. Bell says: "It is a valuable intercurrent in dysentery when Merc. cor., although indicated, fails to relieve." He meant that *Merc. cor.* is *apparently* indicated, but the *mental state* of Aconite is present. Aconite should have been given in the beginning. You can follow with *Merc.*) Aconite very closely resembles *Calc.* in that the diarrhœa comes on from taking cold, but Dulc. has *less pain* and *no fear.* Diarrhœa *with* fear—Aconite. Diarrhœa *from* fear—Gels. A soldier loses his stool involuntarily in battle from fear. Gels. covers this psychical state (see also Arg. nit.). A new-born child has not wet its diaper up to the third day; give Aconite. "Perhaps the child was frightened on coming into the world!" (If a woman fail to micturate after parturition, think of Aconite or Causticum.) If in a patient, perfectly healthy, the menses fail to appear—Sepia. If they appear and stop without cause—Bell., one dose. Child cries violently at night from retention of urine. Frequent desire to urinate from taking cold. Aconite has a suppression of menses from *dry, cold winds,* attended with *fear* of *death* and *anguish.* Labor-like pressure in womb; patient bent double; thirst; great restlessness and irritability. (Suppression of menses from getting feet wet—Puls.) A woman, *without fever,* in the pregnant state, tells you earnestly that she will die during delivery; she is positive of it; one dose of Aconite will cause this mental condition to disappear. Suppression of menses from *fear* without *cold.* I believe Aconite stands at the head of the list for suppression of menses. Croup; waking in *first sleep* in agony; tosses about; loud breathing, every *exhalation* ending in cough. Laryngitis with inflammatory fever. In laryngismus stridulus, Aconite is not the best remedy. There is a rising and wheezing in the larynx; the patient sits up in bed and grasps the throat; the appearance is so frightful that the attendants think the woman will die. You can relieve this in fifteen minutes with either Ignatia, Gels., or Lauroceracus—the tincture will not cure it.

Marked oppression of the chest and short breathing. Angina pectoris, attended with short breathing, excitement, quick, sharp pulse, and especially the *great fear* and *anguish* depicted on the countenance. If patient does not have this *fear* and *anguish,* you may have this oppression with other remedies (Bry., Ars.).

Dry cough, with a hoarse, rasping, rattling in the chest. This is not the rattling of mucus, but spasmodic; not gurgling, like Ipec. and Ant-t. In dry, ringing cough Aconite leads all other remedies. Expectoration scanty or bloody; also thick, white mucus. Great remedy for hæmoptysis. A hemorrhage of *bright red blood*, attended with *fear* and *anxiety*. Burning in the lungs from inflammation of the pleuro-mucous membrane in pneumonia and pleurisy; compelled to lie on the back. When the left lung is involved with the pleura, partially suppressed, dry cough; the pains *aggravated* at *night*, brought on from *dry, cold winds*. The little sputa that comes up is tough, thick, and cherry-red, falls into a pan in a round lump—left-sided pneumonia; with these symptoms, in four hours Aconite will cure this condition. Stitches through the chest; can lie only on the back; burning, weight, and pressure under the sternum. Aconite has a very feeble pulse. (The *Stram.* pulse cannot be felt on the *right side.*) Violent stitches in the heart compel patient to rise in bed. (A casual stitch through the heart, *without* the *Aconite violence—Staph.*) Never forget Aconite in inflammation of the spinal cord in children. Important in spinal meningitis (*Opium*, *Bell.*, and also *Gels.*). Spasms of the back in children generally. Aconite, next to *Bell.*, is important in spasms in children. Spinal and heart complaints, with *tingling* in *left hand* and *fingers* (Cactus grand). A great remedy in acute rheumatism, when first taken down; there is shooting, stinging, and darting pains; they drive him to distraction; formication in hand, arm, and fingers; tearing pain in shoulder-joint, with *fear*. (Rheumatism of the *left* shoulder—*Ferrum.* Rheumatism of the *right* shoulder—*Sang.* The pain is in the deltoid muscle in Ferrum. When he attempts to raise his arm the pain increases, like hot iron—this is myalgia. The sanguinaria pain is more in the *joint* than in the *muscle;* it is a dull, aching pain, sometimes tearing. Pain [neuralgia] from the back of neck down the right arm to the forefinger is *Kalmia.* Under *Calc.-phos.* every change of weather to *cold* brings on rheumatism, no matter where located.) Paralysis of wrist, hands icy cold, cold sweat on palms. Now you would not expect to cure this condition with Aconite unless the patient was a nervous one; you examined the spine and found sensitive spots, and the patient seemed frightened in a crowd; but you must follow it with Sulphur, which is the chronic form of Aconite, so to speak. Rheumatism of the *left* hip—Aconite. Rheumatism of the *right* hip—Sepia. *Hot hands* and *cold feet*—Aconite. *Cold hands* and *hot feet*—Aloe. *Icy cold, blue hands*—Veratrum. Legs and feet feel numb, *commencing* in the *feet* and spreading *upward;* restlessness and continued motion—but not the *relief* from motion we have in *Rhus.* (*Bry.* patient does not want to be moved. Rheumatism from checked diarrhœa—*Abrotanum.* Diarrhœa from rheumatism—*Kali-bich.*) Dreams, anxious and vivid; profuse hot sweat *during sleep.* (*Merc.* has the same symptom, but also *during waking state.*) Aconite patient stops sweating when he wakes. (One will scarcely ever need any other remedies in measles than Aconite, Puls., Sulph., and Euphrasia—Euphrasia for the watery, acrid discharges from the eyes and nose; Puls. if the case was mild and the rash already out; duskiness of the skin. Aconite bright red; the *Aconite* rash is *rough.* Bright red and *smooth* is Bell.)

The Aconite *fever begins* in the *head* and *goes down;* the *cold begins* in the *feet* and *comes up.* Aconite is the remedy for scarlet rash. In worm-fever, with *fear* and *anguish*, after fever has passed off, select constitutional remedy, such as Sulph., Calc., or Cina; correct the condition of the stomach, and the worms will be expelled. Little itching spots like flea-bites; rash-like erythema coming on from being in the sun; dryness and burning of the skin. Little babies, who have jaundice soon after they are born, follow it with Sulph. (I always give a child a few days old one dose of *Sulph.;* it is an anti-psoric.) Persons leading sedentary lives; persons with tonicity of fibre; dark hair and eyes. Aconite antidotes Bell.; *never* alternate them. The abuse of Aconite calls for Sulphur. If you have given a patient Sulphur high, and he takes cold, the symptoms most likely will call for Aconite; give it in the third, because Aconite *low* will cure the *acute* symptoms, and at the same time will not antidote the Sulphur *high.* After he gets over the acute attack, don't interfere with the Sulphur. If you spoil a chronic case, you should let all the drug's effects pass off before prescribing again.

