# Errata

**Published:** 1885-04

**Authors:** 


Errata.—In February number of this journal occur the following errors: Page 70, lines nine and twelve from bottom, for *spasms* read *sepsis;* page 71, line seventeen from bottom, for *Ferrum, Phos.*, read *Ferr-phos.*, and on same line, *add* after Magn-phos., better from heat applied; page 73, second line, for *pleuro-mucous* read *broncho-mucous.*

